# International Consensus Recommendations of Diagnostic Criteria and Terminologies for Extranodal Extension in Head and Neck Squamous Cell Carcinoma: An HN CLEAR Initiative (*Update 1*)

**DOI:** 10.1007/s12105-025-01753-7

**Published:** 2025-02-07

**Authors:** Ruta Gupta, Timothy Fielder, Munita Bal, Simion I. Chiosea, Jane E. Dahlstrom, Aanchal Kakkar, Katalin Kiss, Jan Laco, Neha Mittal, Sunil Pasricha, Spinder Samra, Nina Zidar, Martin Bullock, Rebecca Chernock, William Faquin, Shao Hui Huang, Jean Yang, Sun Och Yoon

**Affiliations:** 1 Department of Tissue Pathology and Diagnostic Oncology, Royal Prince Alfred Hospital, NSW Health Pathology, Sydney, NSW Australia; 2 Faculty of Medicine and Health, University of Sydney, Sydney, NSW Australia; 3 NHMRC Center of Research Excellence for Applied Innovations in Oral Cancer, Sydney, NSW Australia; 4 Department of Pathology, Tata Memorial Center, Homi Bhabha National Institute, Mumbai, Maharashtra India; 5 Department of Pathology, University of Pittsburgh, Pittsburgh, Pennsylvania USA; 6 ACT Pathology, Canberra Health Services, Canberra, Australia; 7 School of Medicine and Psychology, ANU College of Science and Medicine, Canberra, Australia; 8 Department of Pathology, All India Institute of Medical Sciences, New Delhi, Delhi India; 9 Department of Pathology, Rigshospitalet, Copenhagen University Hospital, Copenhagen, Denmark; 10 The Fingerland Department of Pathology, University Hospital Hradec Kralove, Hradec Kralove, Czech Republic; 11 The Fingerland Department of Pathology, Charles University Faculty of Medicine in Hradec Kralove, Hradec Kralove, Czech Republic; 12 Department of Pathology, Rajiv Gandhi Cancer Institute and Research Centre, New Delhi, India; 13 Department of Pathology, NSW Health Pathology, Westmead Hospital, Sydney, NSW Australia; 14 Institute of Pathology, Faculty of Medicine, University of Ljubljana, Ljubljana, Slovenia; 15 Department of Pathology, Faculty of Medicine, Dalhousie University, Halifax, Nova Scotia Canada; 16 Department of Pathology and Immunology, Washington University School of Medicine, Saint Louis, MO USA; 17 Department of Anatomic and Molecular Pathology, Massachusetts General Hospital, Harvard Medical School, Boston, MA USA; 18 Department of Radiation Oncology, Princess Margaret Cancer Centre / University of Toronto, Toronto, ON Canada; 19 Department of Mathematics and Statistics, University of Sydney, Sydney, Australia; 20 Department of Pathology, Yonsei University College of Medicine, Severance Hospital, Seoul, Korea; 21 Tissue Pathology and Diagnostic Oncology, Royal Prince Alfred Hospital| NSW Health Pathology, Building 12, Missenden Road, Camperdown, NSW 2050 Australia; 22 University of Sydney, Central Clinical School, Royal Prince Alfred Hospital, Building 12, Missenden Road, Camperdown, NSW 2050 Australia

**Keywords:** Diagnosis, Extranodal extension (ENE), Histology, Lymph node, Head and neck, Macroscopy, Matting, Soft tissue deposit, Squamous cell carcinoma

## Abstract

**Purpose:**

Extranodal extension (ENE) increases the risk of recurrence and death in head and neck squamous cell carcinoma (HNSCC) patients and is an indication for treatment escalation. Histopathology forms the mainstay of diagnosing ENE. There is substantial variation in the diagnosis of ENE and related terminology. Harmonising the diagnostic criteria for ENE was identified as a priority by the Head and Neck Consensus Language for Ease of Reproducibility (HN CLEAR) Steering Committee and its global stakeholders.

**Methods:**

An international working group including 16 head and neck pathologists from eight countries across five continents evaluated whole slide images of haematoxylin and eosin-stained sections depicting potential diagnostic problems through nine virtual meetings to develop consensus guidelines.

**Results:**

ENE should be diagnosed only when viable carcinoma extends through the primary lymph node (LN) capsule and directly interacts with the extranodal host environment with or without desmoplastic stromal response. Identifying the original LN capsule and reconstruction of its contour can assist in the detection and assessment of ENE. The term matting is recommended for confluence of two or more nodes due to histologically identifiable tumour extending from one LN to another. Matting constitutes major form of ENE. On the other hand, the terms fusion/adhesion/confluence/conglomeration and other synonyms of adhesion should be limited to confluence due to fibrosis or inflammation without histologically identifiable tumour between involved lymph nodes. Tumour extension along narrow needle tracks or spillage of cyst contents following an FNA do not constitute ENE.

**Conclusions:**

The consensus recommendations encompassing the definition of ENE, macroscopic and histologic examination of lymph nodes (LN) and practical guidelines for handling challenging cases are provided.

## Introduction

Extranodal extension (ENE) increases the risk of recurrence and death in patients with head and neck squamous cell carcinoma (HNSCC) [1–4].

The American Joint Commission on Cancer (AJCC) and the Union for International Cancer Control (UICC) recommends upstaging of head and neck cutaneous and Human Papillomavirus (HPV)-independent mucosal HNSCC with ENE [5]. ENE may potentially influence the staging parameters for HPV-associated oropharyngeal squamous cell carcinoma (OPSCC) in the future [6, 7]. ENE is an indication for treatment escalation in HNSCC with the addition of adjuvant radiotherapy or chemoradiation [8, 9].

Histopathology forms the mainstay of diagnosing ENE [5, 10]. There is substantial variation in the diagnosis of ENE and related terminology used amongst pathologists despite the significant treatment ramifications with increased chemoradiation. Classification systems to quantify ENE have been proposed [11, 12] yet interobserver variability remains a challenge [11, 13]. Harmonising the diagnostic criteria for ENE was identified as a priority by the Head and Neck Consensus Language for Ease of Reproducibility (HN CLEAR) Steering Committee and its global stakeholders. An international working group including 16 head and neck pathologists, a radiation oncologist, a surgeon and a statistician from eight countries across five continents was formed as per the HN CLEAR requirements for a Working Group with subject matter expertise [14].

The working group adopted a combination of Nominal Group Technique and Consensus Development Conference methods [15]. The first phase included systematic review of the literature as per the Preferred Reporting Items for Systematic Reviews and Meta-Analyses (PRISMA) yielding limited studies addressing the diagnostic criteria for ENE [16–18]. Each pathologist in the working group then submitted whole slide images of haematoxylin and eosin-stained sections from four-five examples of nodal metastases in which potential issues or conceptual considerations rendered the diagnoses of ENE challenging. The initial virtual discussions focussed on identifying the causes underlying the diagnostic challenges and ensuring that all challenges faced by pathologists in their routine work related to diagnosing ENE were included. Nine virtual meetings were dedicated to developing consensus recommendations for resolving these issues using the scanned whole slide images. The histologic concepts and findings discussed by the working group were not previously discussed in the literature. The proceedings of all meetings were recorded in the form of a working email trail that was used by all participants to refine the consensus recommendations and develop the guidelines.

The consensus recommendations encompassing the definition of ENE, macroscopic and histologic examination of lymph nodes (LN)s are presented below. Practical guidelines for handling challenging cases are provided.

### Definition

Extranodal Extension: Extension of viable tumour outside the LN capsule into the perinodal soft tissues with or without a desmoplastic reaction [19].

**Fig. 1:**
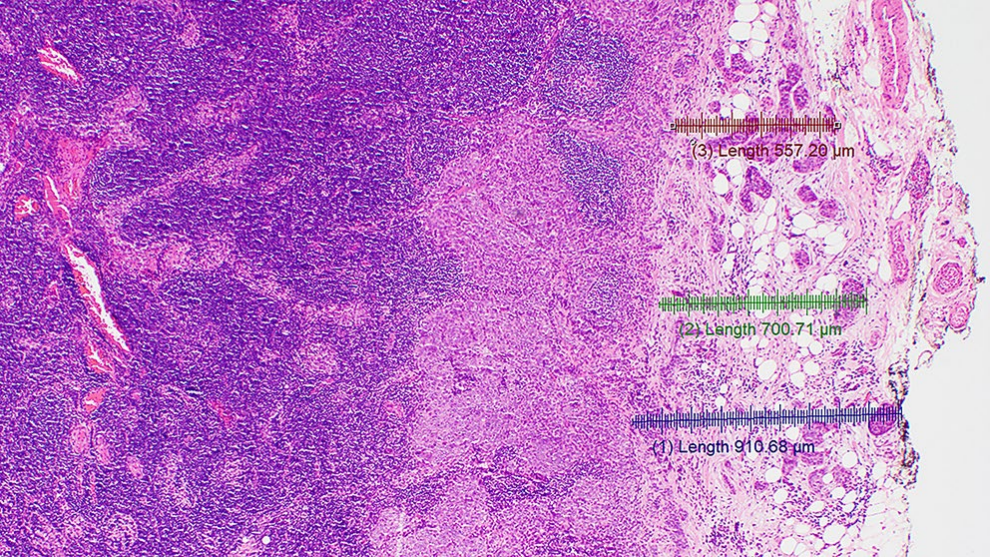
LN with ENE with tumour nests infiltrating beyond the lymph node capsule. The tumour nests are closely associated with fibroadipose tissue and medium calibre blood vessels (HE 10X). The blue line demonstrates the maximum extent of ENE that is included in the pathology report (approximately 1 mm)

ENE indicates that the tumour has infiltrated beyond the LN capsule and can directly interact with the extranodal host microenvironment (Fig. 1).

### Macroscopic Examination to Maximise the Detection and Assessment of ENE

- Often, the surgical teams submit elective neck dissection specimens from clinically/macroscopically uninvolved lymph nodes as multiple specimens separated by neck lymph node groups (Levels IA, IB, IIA, IIB, III, IV and V). These specimens are best served by blunt dissection to retrieve the lymph nodes. Without appropriate orientation, margin assessment of any occult metastasis with ENE detected in these nodes is unreliable.
- Macroscopically uninvolved LNs should be submitted entirely for histologic examination [19].
  - LNs *≤* 5 mm can be submitted whole.
  - LNs > 5 mm can be multisected perpendicular to the long axis to achieve slices 2–3 mm in thickness.
- For all LNs, leaving small amounts of fibroadipose tissue attached to the LN capsule assists in detection and assessment of minor ENE.

**Fig. 2:**
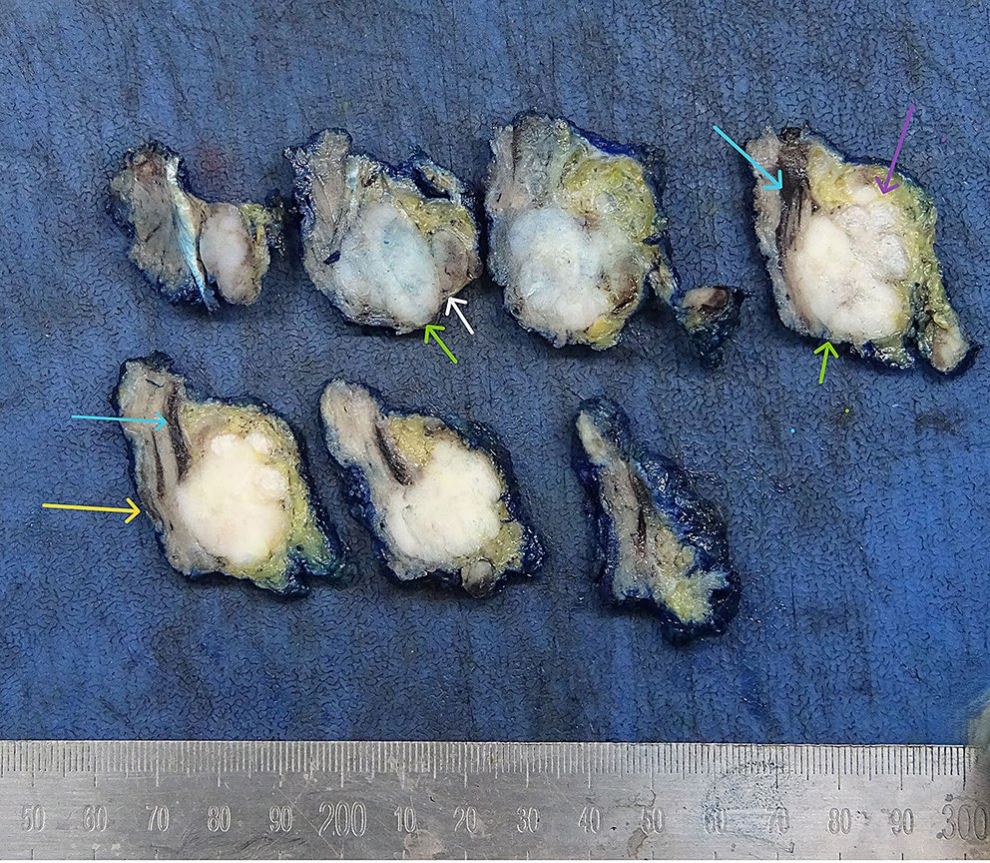
Demonstrates the macroscopic examination of an involved lymph node: The node is serially sliced at 3 mm interval. The slices are laid out sequentially and photographed. The sections are selected to demonstrate the lymph node with metastatic deposit (white arrow) and the relation of the metastases to the internal jugular vein (Blue arrow), the sternocleidomastoid (yellow arrow) and confluence of multiple nodes (purple arrow) and the closest potential margin (Green arrows). Institutions with access to megablock/sections can embed 1–2 sections demonstrating the greatest extent of ENE with closest margins

The examination of macroscopically involved LNs should be optimised for identifying ENE, its greatest extent, involvement of attached structures and proximity to the potential soft tissue margin. This is best achieved by serially sectioning the specimen (Fig. 2):

1. The size of the metastases is best measured macroscopically for obviously involved LNs.
2. Inking of the external surface is recommended by The International Collaboration on Cancer Reporting (ICCR) recommendations [19] and should follow institutional preferences of the head and neck multidisciplinary team (HNMDT).
3. Serially section perpendicular or parallel to the long axis of the LN.
4. Lay out the serial slices for visual inspection.
5. Macroscopic photographs for documentation also facilitate decisions regarding submission of more sections following histologic examination.
6. Select the areas with macroscopically greatest extent of ENE, infiltration from the LN into adjacent structures and closest margins for histologic examination (Fig. 2).
7. For radical and modified neck dissections, the interface of the positive LN with the jugular vein (Blue arrows) sternocleidomastoid (SCM) (Yellow arrow) and/or, accessory spinal nerve should be sampled.
8. In case of confluent LNs, the interface between the LNs should be sampled for histologic evaluation (white arrows).

### Histologic Evaluation of ENE

ENE should be diagnosed only when the tumour transgresses the entire thickness of the original (normal/primary) capsule of the LN (Fig. 1; Table 1).

**Table 1:** Criteria for diagnosis of ENE: HNCLEAR recommendations (update 1)

**Positive for ENE**
Complete transgression of the primary LN capsule with extension into the perinodal soft tissues. Infiltration into reduplicated LN capsule. Infiltrative tumour nests surrounded by adipocytes on multiple sides in the LN hilum. Matted LNs with histological evidence of tumour crossing from one LN into another.
Presence of viable tumour cells in perinodal soft tissues directly interacting with the extranodal host microenvironment in patients with neoadjuvant therapy.
**Negative for ENE**
Tumour Confined within the LN capsule. Thickened LN capsule without intracapsular viable tumour cells. Tumour contained within the LN focally juxtaposed to the hilar adipose tissue. Tumour extending along a linear FNA or core biopsy tract. Fusion or adhesion of lymph nodes without histological evidence of tumour crossing from one LN into another. Spilled necrotic debris or cyst content without viable cells. Tumour confined within blood vessel walls (Lymphovascular emboli). Tumour within or around nerves (Perineural involvement).

#### Spatial Reconstruction of the LN Capsule and Contour

**Fig. 3:**
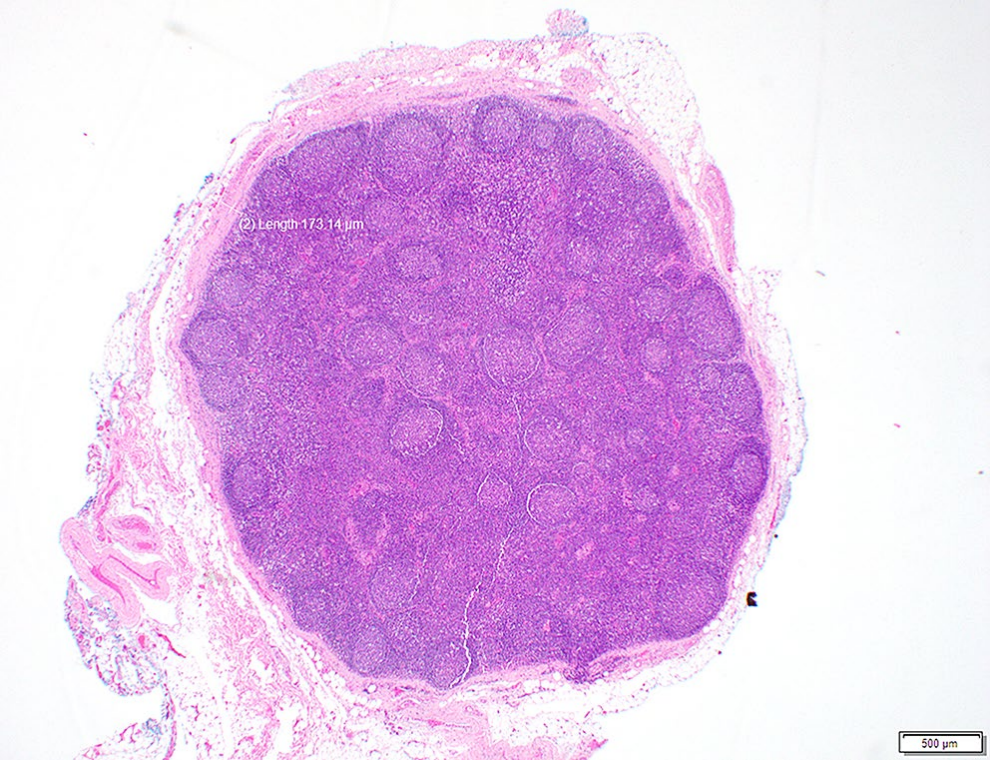
Shows a normal lymph node with its original thin fibrous capsule measuring approximately 0.2 mm in thickness (HE 4X)

The original LN capsule should be used as a guide to trace the LN contour. It is easier to identify the contour and the thickness of the original LN capsule adjacent to the area with ENE rather than within the ENE. In the absence of unequivocal original capsule on the tissue section of interest, one could refer to normal or benign LNs from the patient’s neck dissection. Generally, the LN capsule is a thin fibrous structure ranging in thickness from approximately 0.1 to 0.5 mm (Fig. 3).

**Fig. 4:**
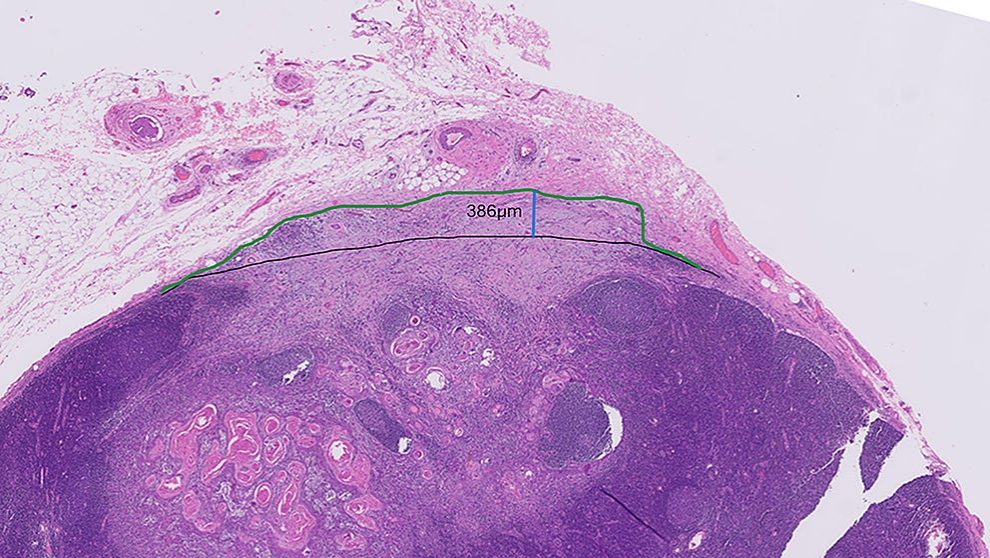
Shows spatial reconstruction of the original/normal/primary lymph node capsule (black line) and the contour of the ENE (green line) (HE 4X). The blue line indicates the measurement of ENE (approximately 0.4 mm). Note the abrupt angulation of the green line; such abrupt distortion in the LN contour can assist with spotting minor ENE

- The LN contour can be spatially reconstructed by identifying the LN capsule (Fig. 4, black line) on both sides of the potential focus of ENE (Fig. 4, green line).

- The line connecting normal capsule on both sides of the potential ENE also serve as a reference line from which to measure the extent of ENE.
- Adipose tissue and blood vessels generally lie external to the LN capsule and the subcapsular sinus. These can be used as a guide to the external most limit of the nodal capsule.

#### Reduplication of the LN Capsule

**Fig. 5:**
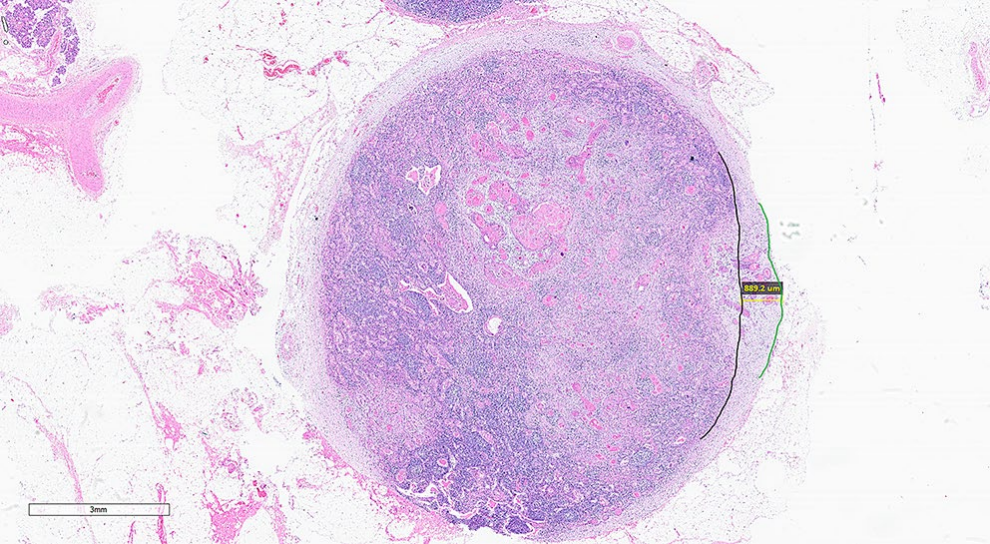
Shows a lymph node with reduplicated thickened capsule. The black line represents the most likely innermost aspect of the reduplicated capsule and the original lymph node contour and capsule. The green line represents the outermost aspect of the reduplicated capsule and ENE. The extent of ENE can be measured between the black and green lines (yellow line) and is approximately 0.9 mm in this section (HE 4X)

Host response can produce capsular reduplication without identifiable original residual capsule (Fig. 5). In these instances, the original capsule can be presumed to be the innermost aspect of the thickened capsule closest to the lymph node parenchyma. The transgression of the presumed original capsule (Fig. 5, black line) by the tumour constitutes ENE (Fig. 5, green line).

#### ENE at the LN Hilum

**Fig. 6:**
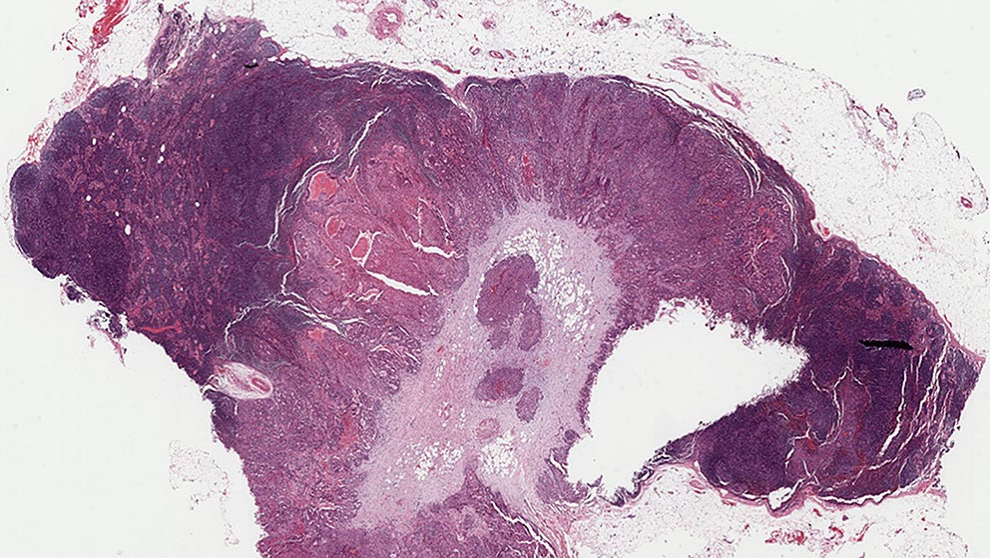
Shows ENE at nodal hilum. There are variably sized irregular nests of squamous cell carcinoma (SCC) surrounded on multiple sides by hilar adipose tissue. Associated desmoplastic stromal response is also present (HE 4X)

The capsule is often incomplete at the LN hilum and the lymphocytes may be closely juxtaposed to the hilar adipose tissue. Several thick-walled blood vessels may be present in this area. Nests of tumour cells infiltrating through the hilar adipose tissue or surrounded by the hilar adipose tissue on more than one side constitute ENE at the hilum (Fig. 6). This is distinct from a predominantly intranodal tumour partially juxtaposed to the hilar adipose tissue due to a deficient capsule. A desmoplastic stromal response, if present, can also be helpful (Fig. 6).

#### Post Neoadjuvant Therapy ENE

**Fig. 7:**
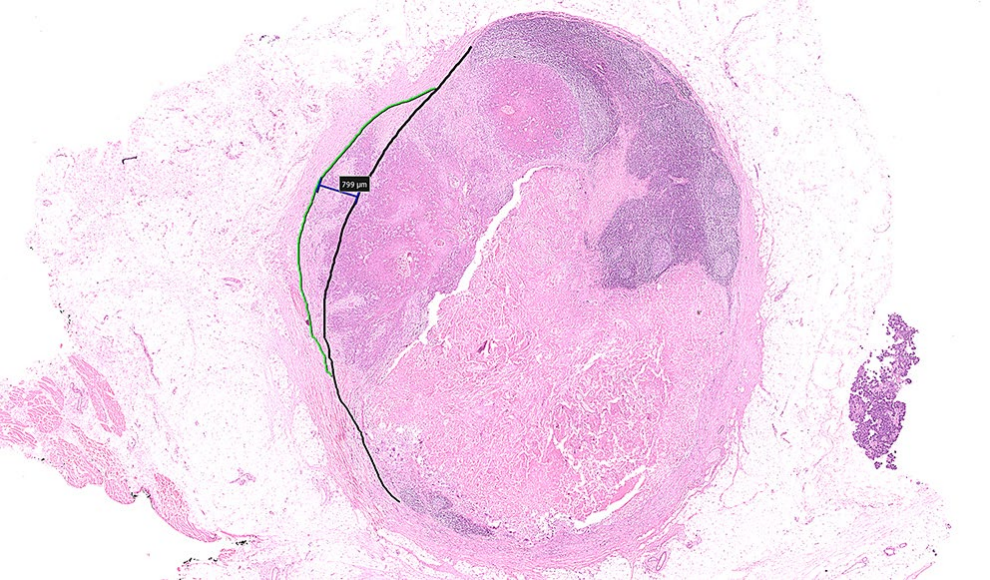
Shows a LN from a patient with neoadjuvant chemotherapy (NACT) showing approximately 40% residual viable tumour. The viable tumour is seen transgressing the original/normal capsule (black line) of the lymph node. The green line depicts the contour of the reduplicated capsule and the blue line the extent of ENE (HE 1.25X)

In patients with neoadjuvant radiotherapy and/or chemotherapy, only viable carcinoma that has completely transgressed through the LN capsule into the adjacent tissues with or without stromal reaction constitutes ENE (Fig. 7).

### Measurement of ENE

The extent of ENE may carry different prognostic significance in HNSCC [12, 20]. Thus, the ICCR structured pathology report includes minor ENE (*≤* 2 mm) and major ENE (> 2 mm) as core elements [19]. Various other international pathology reporting protocols including those by the College of American Pathologists (CAP), the Royal College of Pathologists of Australasia (RCPA) recommend measurement in millimeters as well as recognise thresholds of 1–2 mm.

1. The extent of ENE is measured perpendicular to the capsule (and not horizontally) and reported in millimetres.
2. Most commonly this includes the thickness of reduplicated LN capsule.
3. Precise measurement of distance is influenced by multiple factors:
  - Block selection at the time of macroscopic examination.
  - Level of the block provided by the microtomist.
  - Contour of the original capsule traced by the pathologist.
4. The above factors make distinction between thresholds of 1–2 mm or greater difficult.

### New Terminology

Physical confluence of two or more LNs can occur due to a variety of reasons. The prognostic significance of LNs matted due to histologically identifiable tumour extending from one LN into another is well established. On the other hand, the prognostic significance of LNs adherent to each other due to inflammation or desmoplasia without histologically identifiable tumour extending from one LN into another requires further evaluation. Inappropriate, interchangeable use of matting with fusion, adhesion and similar terms, renders data accrual and analyses difficult. Thus, separating the term matting - used to indicate weaving of tumour from one lymph node into another from fusion, adhesion and similar terms (e.g. confluence, conglomeration) will ensure adoption of uniform terminology globally.

Prospective studies will be required to ascertain the prognostic significance of distinguishing matting from other terminology. The definitions recommended are:

#### Matting

**Fig. 8:**
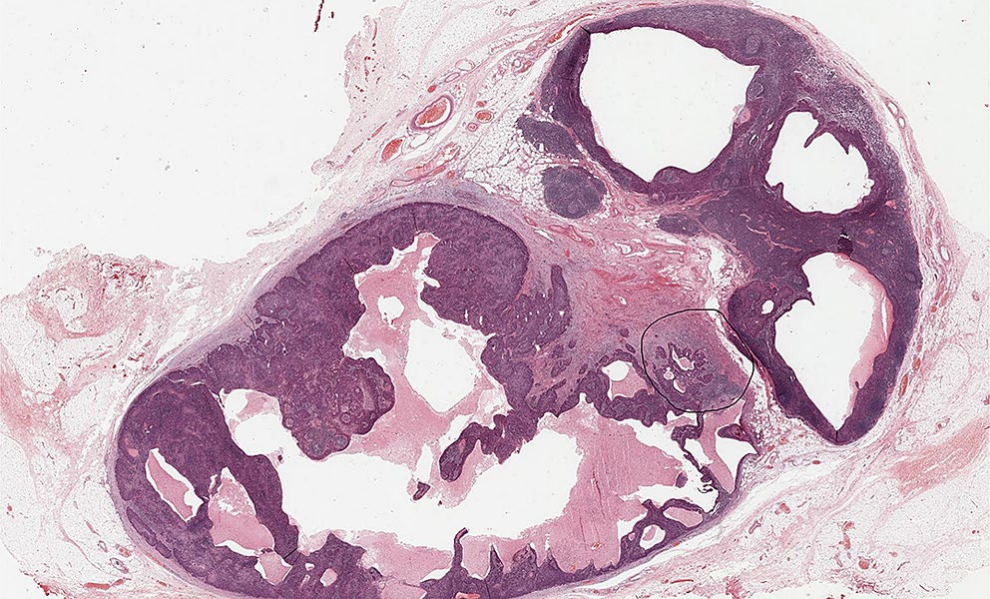
Demonstrate matted lymph nodes with tumour nests and desmoplastic stromal response extending from one lymph node to another (Black circle). (H&E, 2x)

**Fig. 9:**
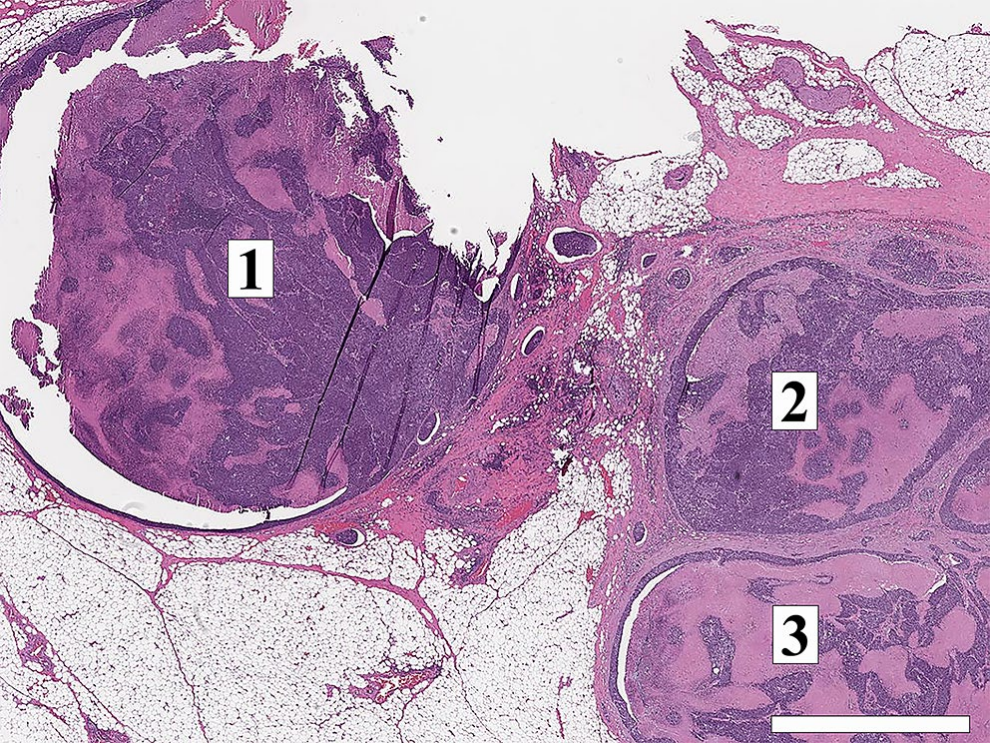
Demonstrate three matted lymph nodes with tumour nests and desmoplastic stromal response extending from one lymph node to another. Scale bar is 2 mm. (H&E, 2x)

Two or more LNs stuck to each other because of histologically identifiable viable tumour extending from one LN into another (Figs. 8 and 9).

#### Confluence/Conglomeration/Fusion/Adhesion

**Fig. 10:**
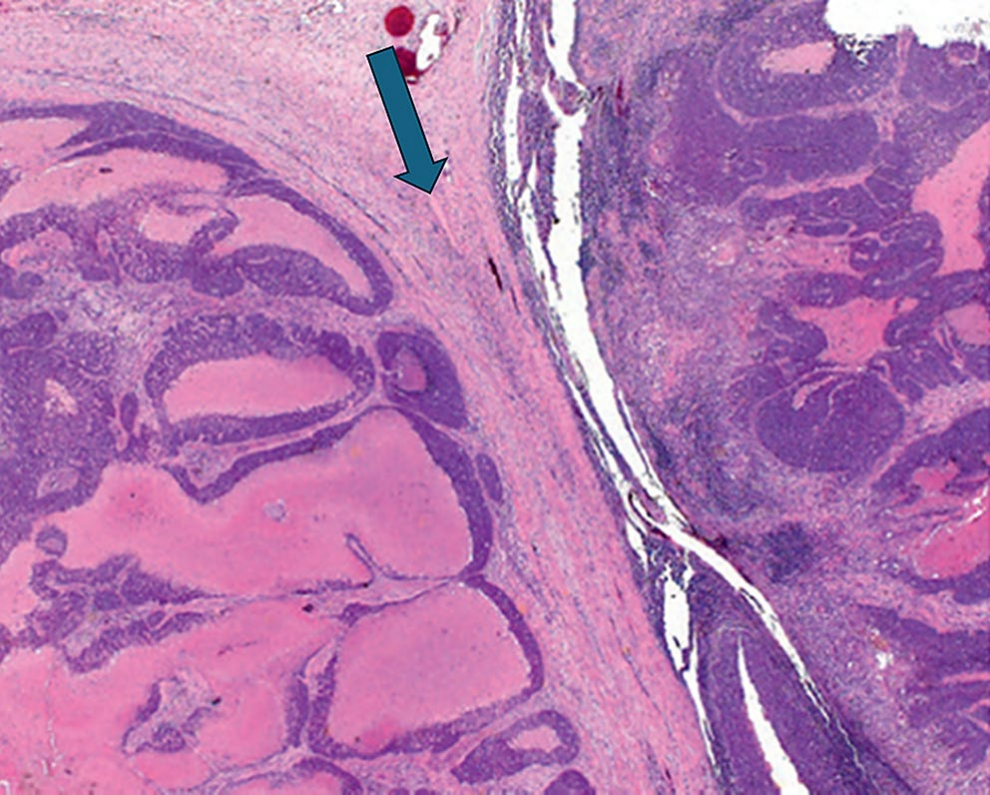
Demonstrate lymph nodes adherent to each other with thickened capsules. However, tumor is not seen transgressing either capsule

**Fig. 11:**
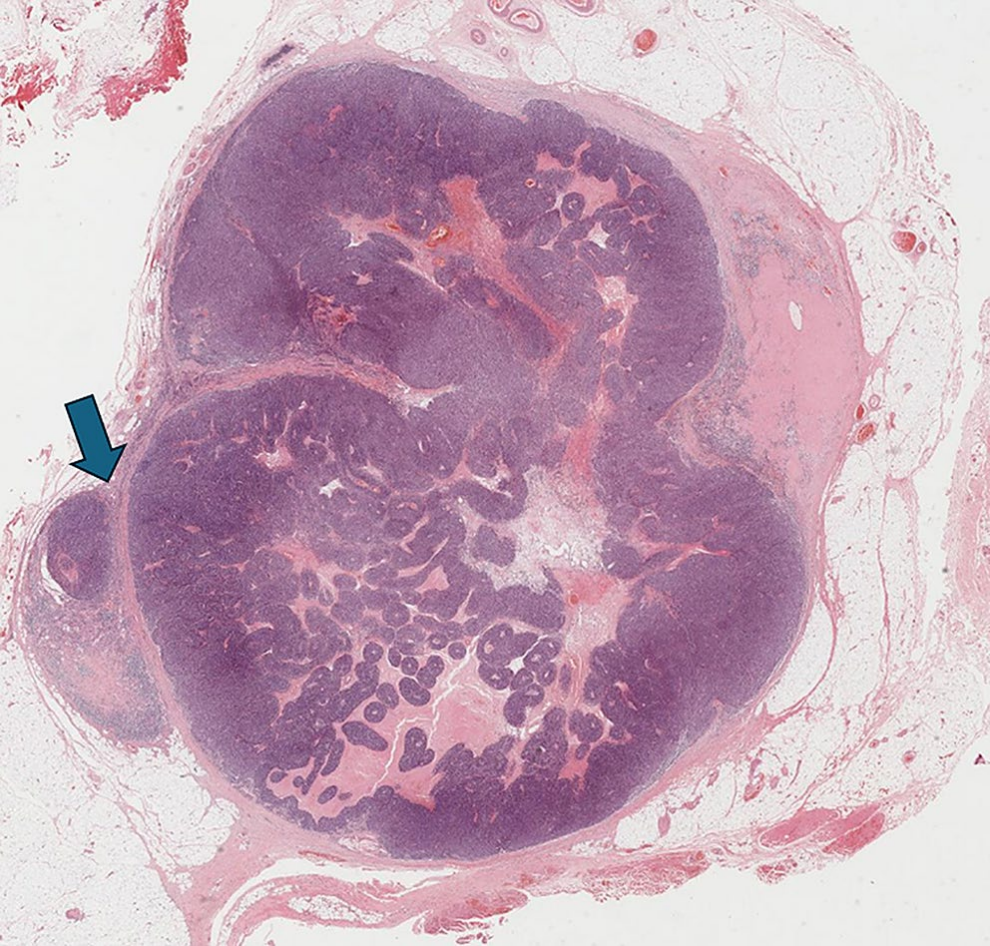
Demonstrate lymph nodes adherent to each other with thickened capsules. However, tumor is not seen transgressing either capsule

Two LNs adherent to each other due to inflammation or thickening of their capsules without histologic evidence of tumour crossing the tissue between the LNs (Figs. 10 and 11). This is not considered as ENE.

All due diligence (macroscopic assessment of the adherent LNs, submitting the interface between involved lymph nodes entirely for histologic evaluation, and examination through levels) should be performed prior to the designation of fusion or adhesion. Presence of adhesion can be included in clinical reports to allow data collection for prospective studies evaluating its prognostic significance. However, the use of the terms “confluence/conglomeration/fusion/adhesion” and their synonyms in clinical reports should be accompanied by a statement on ENE being absent or indeterminate.

### Soft Tissue Tumour Deposit

**Figs. 12:**
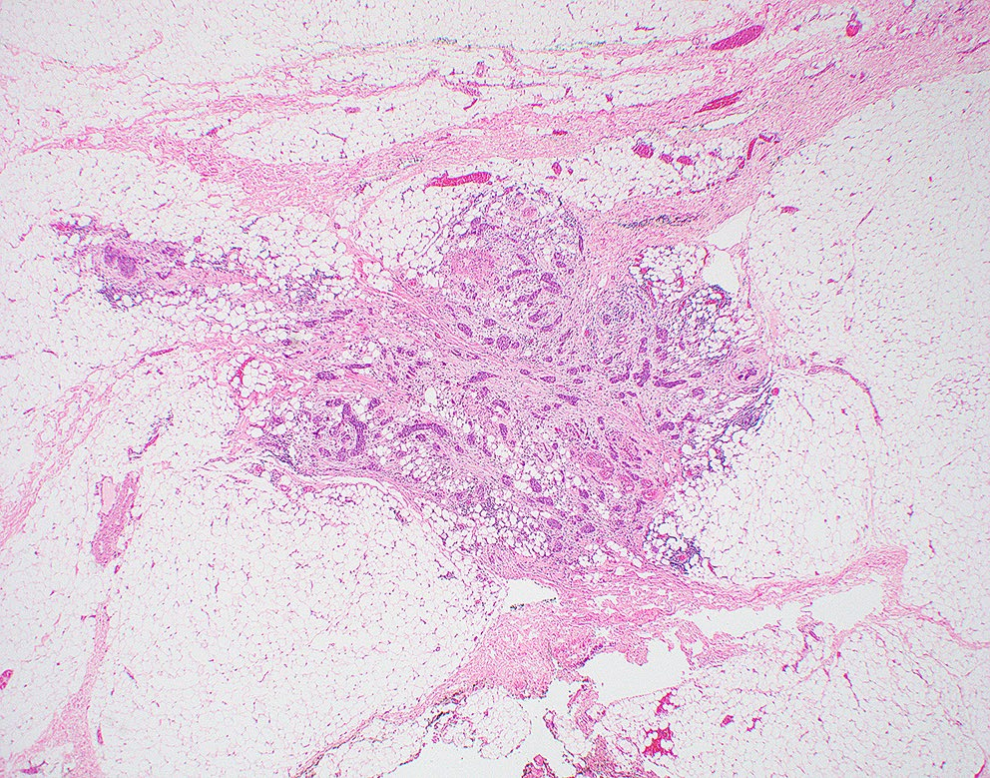
Demonstrate soft tissue deposits without discernible lymph node structure (HE 1.25X)

**Figs. 13:**
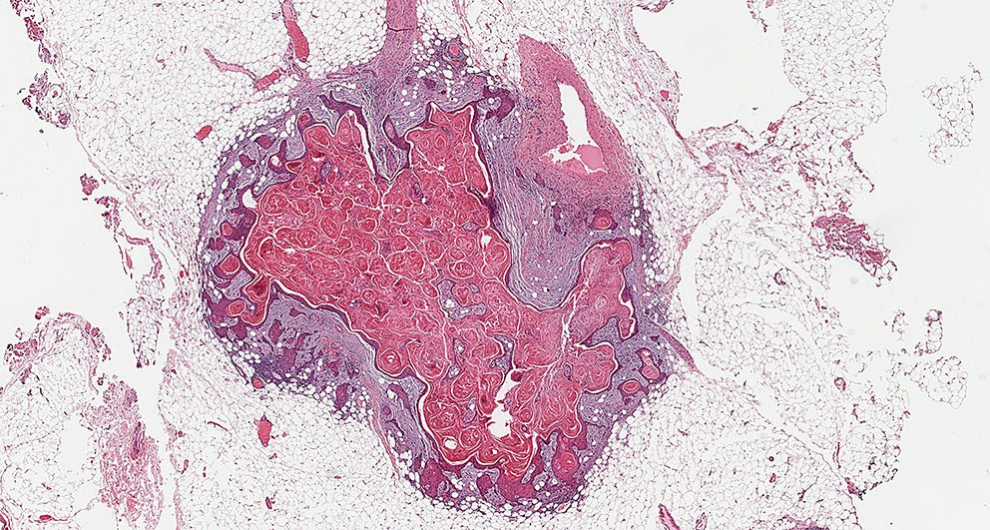
Demonstrate soft tissue deposits without discernible lymph node structure (HE 1.25X)

An irregular or rounded deposit of tumour within soft tissues without a discernible residual LN capsule, subcapsular sinuses or germinal centers (Figs. 12 and 13).

Soft tissue deposit occurs in the context of complete effacement of a LN by the tumour or tumour extending out of a blood vessel or lymphatic directly into the connective tissues.

#### Criteria for identifying soft tissue tumour deposits

- No minimum size criterion.
- No discernible residual LN structure.
- Should be in the primary cancer’s lymphatic drainage area.
- Should be discontinuous from the primary tumour.
- Number of deposits should be recorded and contribute to the pN staging as positive lymph nodes with major ENE.

#### Factors that should not be seen in soft tissue tumour deposits

- Histological evidence of a residual LN *constitutes LN metastasis with ENE*.
- Tumour deposit identified within and still confined to lymphatic or vessel lumen, *constitutes lymphovascular invasion* (Table 1).
- Tumour deposit identified within or around a nerve *constitutes perineural invasion* (Table 1).

### Measurement of Matted LNs and Soft Tissue Tumour Deposits

1. Size of a macroscopically obvious matted or soft tissue tumour deposits > 15 mm is best measured during macroscopic examination (Fig. 2).
2. Involvement of structures such as internal jugular vein or SCM should also be documented during macroscopic examinations as this constitutes major form of ENE. Sections should be taken to demonstrate involvement of these structures when possible.
3. It can be difficult to count the number of matted LNs. Correlation with macroscopic appearance is useful. Spatial reconstruction of LN contours is also useful. For example: The confluent mass in Fig. 8 includes two LNs, while that in Fig. 9 includes three LNs. Overall, finding of matted LNs implies the presence of at least two positive LNs. If a precise count is not possible, it is useful to state if the conglomerate includes less than or more than three LNs.
4. LN matting constitute major or extensive (> 2 mm) ENE by definition (Figs. 8 and 9).

### When in Doubt About ENE

**Fig. 14:**
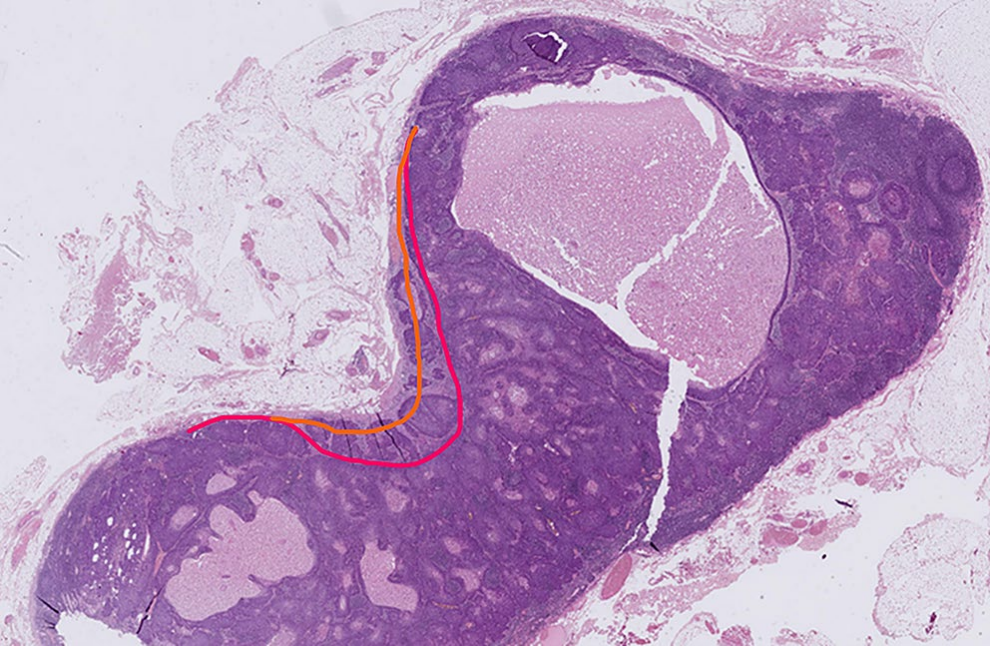
Demonstrates the difficulty in ascertaining the plane of the true capsule (yellow contour vs. pink contour). The measurement of ENE will vary with the contour selected. However, both measurements constitute minor ENE (HE 1.25X)

1. Submit the entire LN capsule for histologic evaluation.
2. Perform levels. While, a definite number of levels is not prescribed, deeper levels should be performed till either the issue is resolved to the satisfaction of the reporting pathologist, or the area of concern is exhausted.
3. Immunohistochemistry with a cytokeratin or p40 can be useful for identifying tumour cells amidst reactive fibroblasts or endothelial cells and can delineate the presence and extent of ENE.
4. Immunohistochemistry with p40 can also be useful in post neoadjuvant therapy samples to identify viable tumour cells in intra- or extranodal location. Of note, non-viable SCC cells also show cytokeratin expression limiting its utility.
5. Radiological/clinical correlation including history of fine needle aspiration (FNA) or core biopsy is useful.
6. Radical or modified radical neck dissections imply significant clinical concern for LNs with ENE. Thus, a result of “negative for ENE”, ideally, should not be rendered without examining the relationship between tumour and SCM, internal jugular vein, and /or spinal accessory nerve.
7. Show a colleague contributing to the head and neck pathology multidisciplinary team discussions.
8. If the situation is not resolved despite best attempts following due diligence- the term ‘indeterminate’ for ENE may be used with a description of the confounding factors. While data are currently not available, the indeterminate category should form a minority (< 5%) of all cases reported by a pathologist.
9. In certain cases, ENE is not in doubt, but it is difficult to precisely measure the extent of ENE (Fig. 14). In such instances the following comment can be used: *Positive for ENE*,* indeterminate for the extent of ENE*,* but at least x mm.*

### What does Not Constitute ENE (Table 1)

**Fig. 15:**
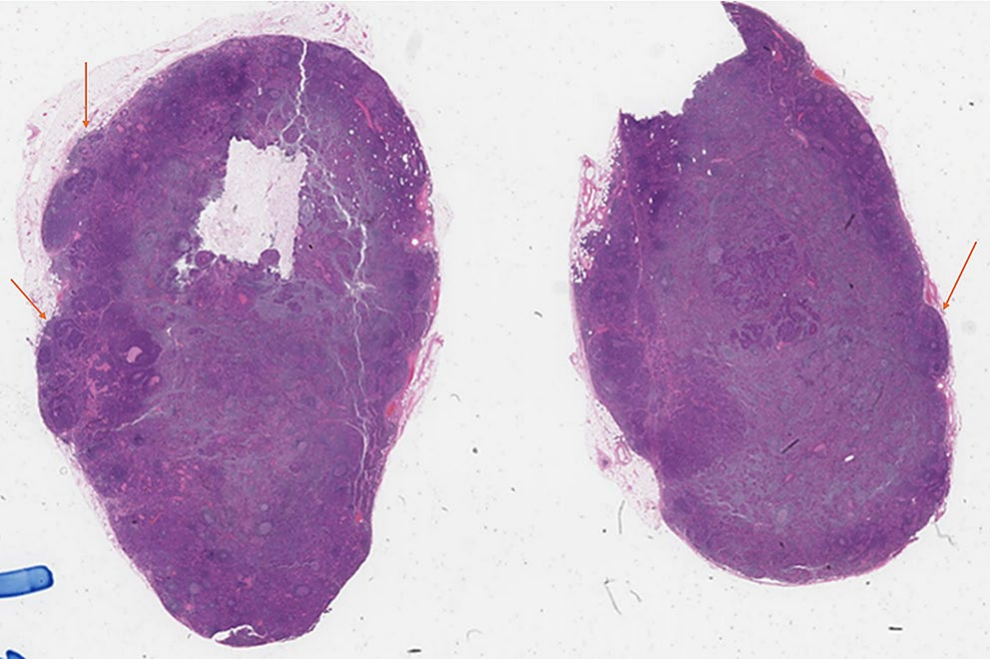
Demonstrates an LN with a lobulated contour, The lobulation is prominent in one half of the lymph node (orange arrows) (HE 1.25X). The mirror image of the lobulated contour is seen in the bisected half and should not be interpreted as ENE

**Fig. 16:**
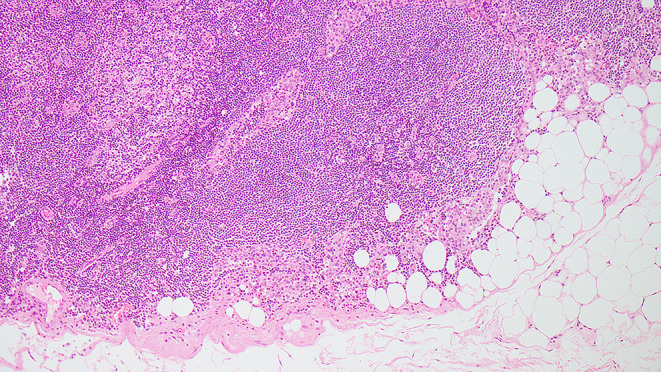
Demonstrates a normal lymph node with subcapsular adipose tissue (HE 4X). Tumour in this region should not be called ENE if the overlying capsule is intact

**Fig. 17:**
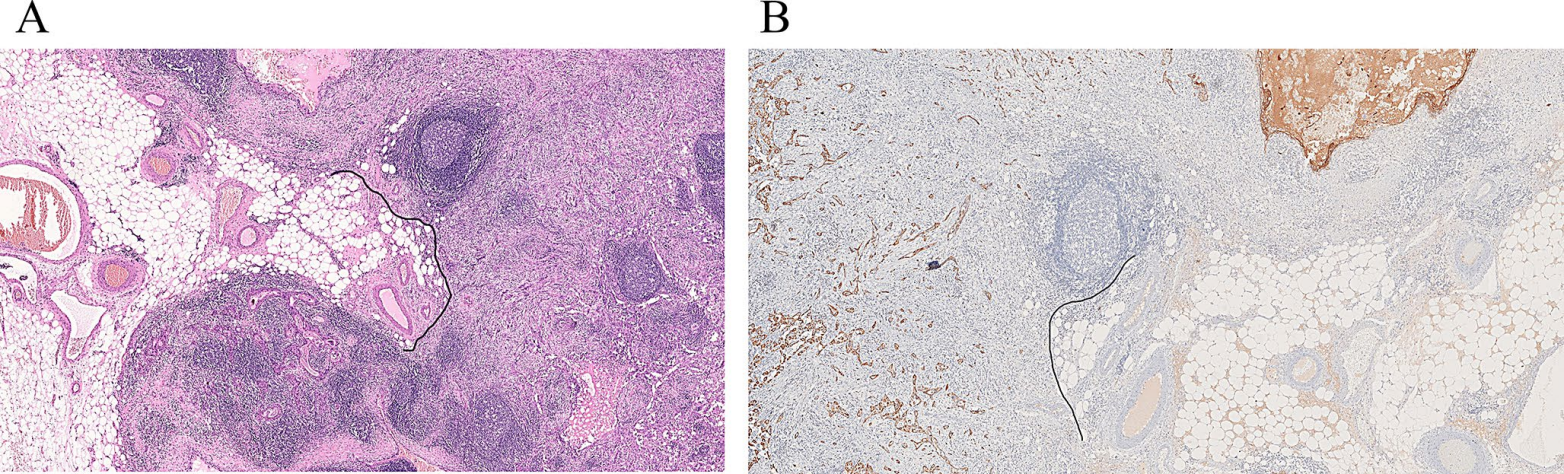
Shows lymphoid parenchyma mingling with large vessels and adipose tissue at the LN hilum (**A**). However, ENE is not present as confirmed by immunostaining for cytokeratin (**B**) (IHC, 4X)

**Fig. 18:**
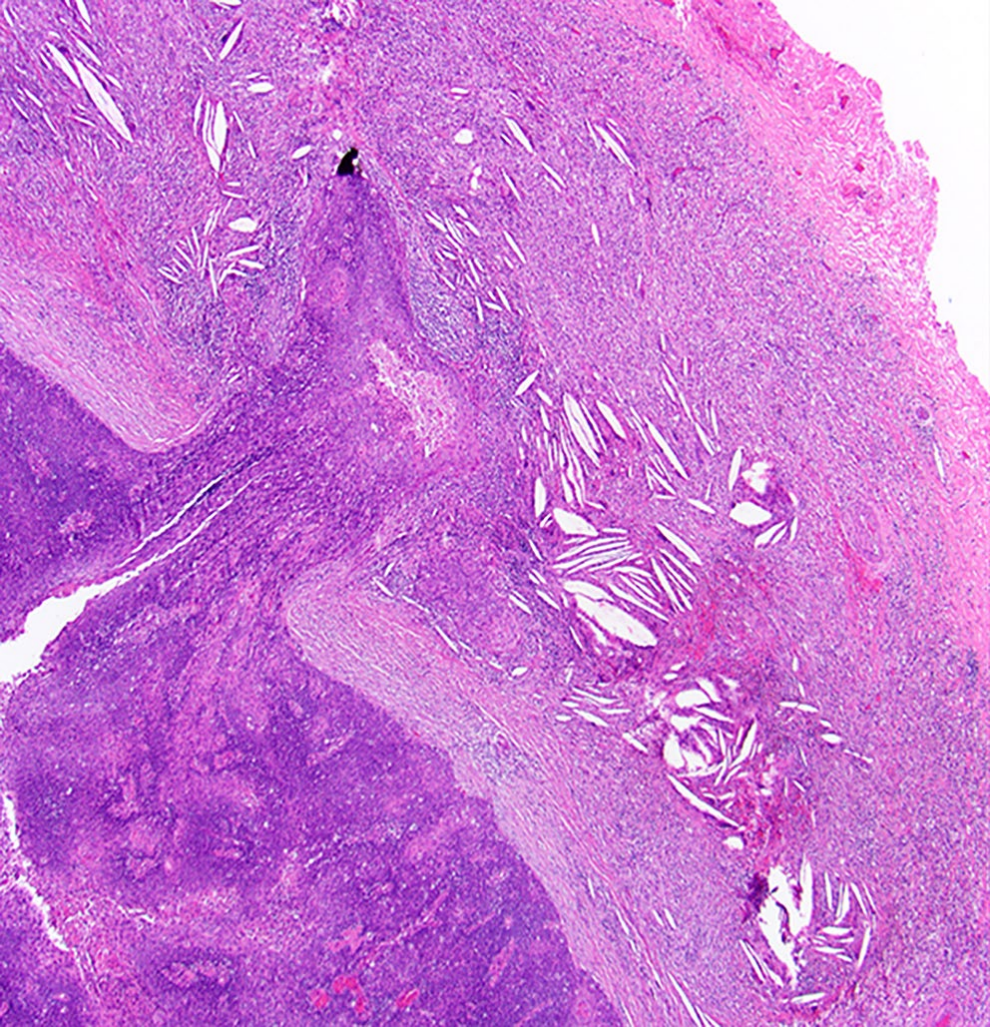
Shows tumour extending through the capsule along a narrow linear tract surrounded by granulation tissue and cholesterol clefts. This is extension along a biopsy tract and should not be considered as ENE (HE 4X)

**Fig. 19:**
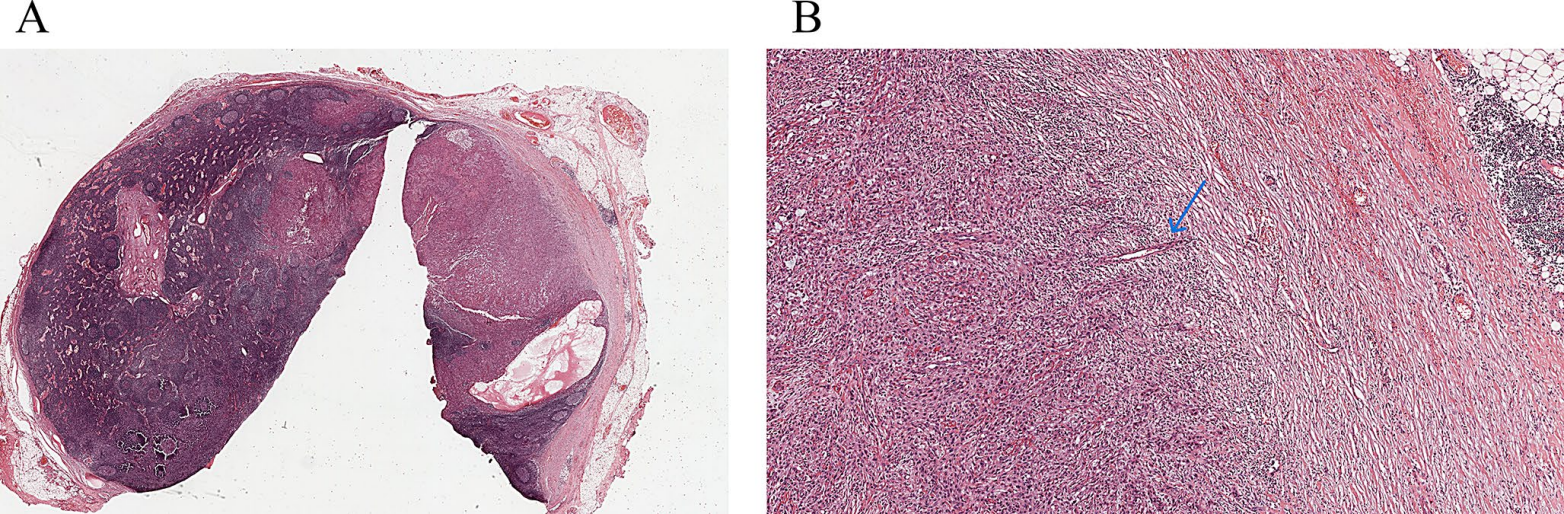
Shows an LN with a previous FNA ((HE 4X) (**A**). There is granulation tissue with thin walled blood vessels aligned parallel to the biopsy tract (HE 10X) (**B**). There is no ENE as tumour is not seen transgressing the thickened capsule

**Fig. 20:**
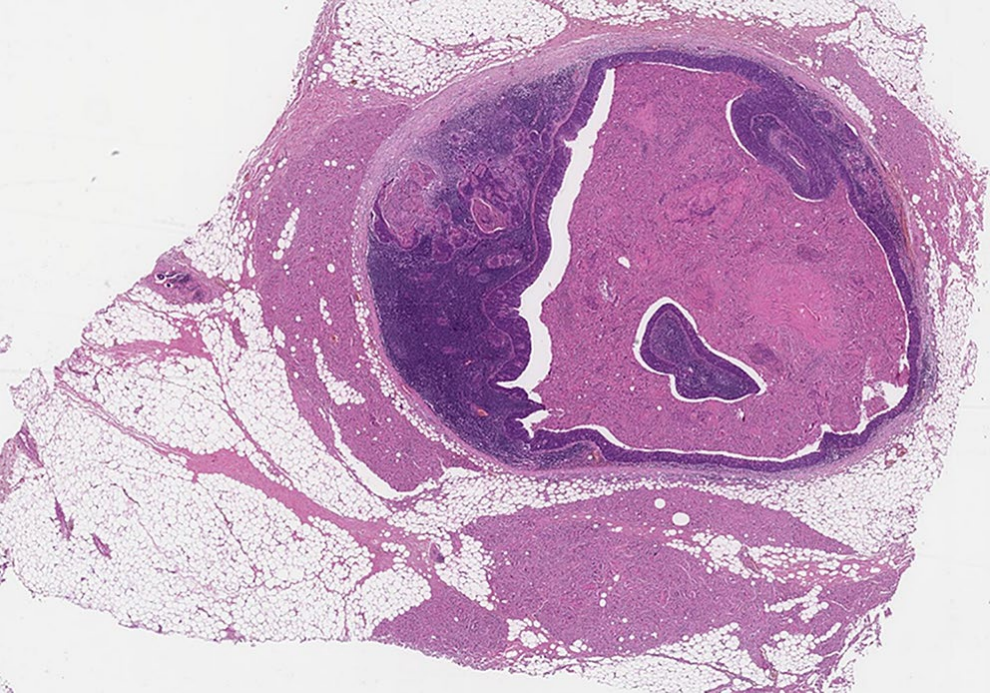
Shows spillage of cyst contents within the perinodal adipose tissue. The rare viable tumour cells surrounded by the necrotic debris within the spillage do not constitute ENE (HE 1.25X)

**Fig. 21:**
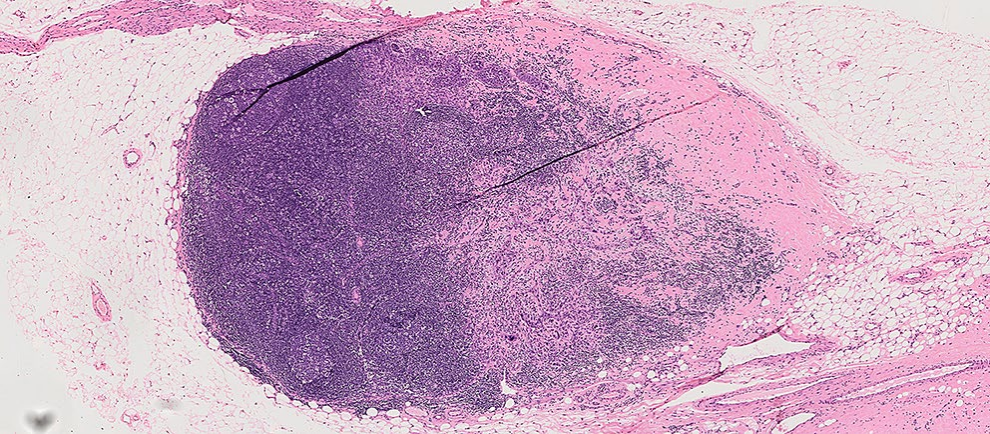
Shows LN from a patient with NACT. The residual viable tumour is confined to the lymph node parenchyma. However, the tumour bed with hyalinisation is causing thickening of the capsule. This should be reported as tumour bed and not as viable ENE (HE 4X)

1. LNs can have lobulated contours (Fig. 15). This contour irregularity should not be interpreted as ENE if the capsule or subcapsular sinuses are intact. In a bisected LN, presence of the mirror image of the lobules/irregular contour in the other half of the LN can be a useful guide for identifying the lobulated pattern (Fig. 15 orange arrows).

2) Tumour within perinodal lymphatics and blood vessels should be described as lymphovascular tumour emboli and not designated as ENE as is also indicated by ICCR [19] and Abou-Foul et al. [17].
3) Occasionally, adipocytes and blood vessels may be present in subcapsular location. ENE should not be diagnosed in these instances if the overlying LN capsule appears intact (Fig. 16).

4) Predominantly intranodal tumour partially juxtaposed to the hilar adipose tissue does not constitute ENE (Fig. 17).

5) Tumour within a biopsy tract does not constitute ENE.
  - A previous FNA or core biopsy tract should be identified by presence of granulation tissue, cholesterol clefts and fibrosis of variable degrees (Fig. 18). Generally, the penetration point of the tumour is linear and narrow reminiscent of a needle tract. In this scenario, even though scanty viable SCC cells are present in the extranodal tissue, it does not constitute ENE.
  - In cases with more remote history of FNA, presence of thin-walled blood vessels perpendicular to the LN capsule (aligned parallel to the tract) can be helpful. Figure 19a demonstrate capsular thickening following an FNA. Tumour is admixed with the granulation tissue and abuts the capsule but does not infiltrate it (Fig. 19b).
  - In the context of a cystic metastasis, FNA may lead to spillage of the cyst contents into the fibroadipose tissue (Fig. 20). The spilled material generally includes keratinous and inflammatory debris. The spilled debris should not be designated as ENE.
  - Scattered viable tumour cells surrounded by the spilled debris should also be considered as a component of the spillage and do not constitute ENE. ENE should be diagnosed only if the viable cells are seen infiltrating and interacting directly with the host tissues and not with histiocytic response to cyst contents.

6) In patients with neoadjuvant treatment, presence of only non-viable tumour cells, anucleate keratinous debris or areas of hyalinization/fibrosis, granulation tissue or macrophages is best considered as post neoadjuvant therapy tumour bed as shown in Fig. 21 and reported as such. This should not be counted as post neoadjuvant therapy ENE.

## Summary

ENE should be diagnosed only when the viable tumour has completely transgressed the primary LN capsule and can directly interact with the extranodal host environment with or without desmoplastic stromal response. Identifying the residual original LN capsule and reconstruction of its contour can assist in the detection and assessment of ENE. The term matting is recommended for confluence of two or more nodes due to histologically identifiable viable tumour extending from one LN to another. On the other hand, the terms fusion/adhesion are recommended for confluence due to fibrosis or inflammation without histologically identifiable tumour. While matting constitutes a major form of ENE, fusion/adhesion is not considered ENE. Stringent criteria should be applied while diagnosing ENE at the LN hilum. Tumour extension along narrow needle tracks or spillage of cyst contents following an FNA do not constitute ENE.

## Data Availability

No datasets were generated or analysed during the current study.
