# Correction: International Consensus Recommendations of Diagnostic Criteria and Terminologies for Extranodal Extension in Head and Neck Squamous Cell Carcinoma: An HN CLEAR Initiative (*Update* 1)

**DOI:** 10.1007/s12105-025-01772-4

**Published:** 2025-03-19

**Authors:** Ruta Gupta, Timothy Fielder, Munita Bal, Simion I. Chiosea, Jane E. Dahlstrom, Aanchal Kakkar, Katalin Kiss, Jan Laco, Neha Mittal, Sunil Pasricha, Spinder Samra, Nina Zidar, Martin Bullock, Rebecca Chernock, William Faquin, Shao Hui Huang, Jean Yang, Sun Och Yoon

**Affiliations:** 1 Department of Tissue Pathology and Diagnostic Oncology, Royal Prince Alfred Hospital, NSW Health Pathology, Sydney, NSW Australia; 2 Faculty of Medicine and Health, University of Sydney, Sydney, NSW Australia; 3 NHMRC Center of Research Excellence for Applied Innovations in Oral Cancer, Sydney, NSW Australia; 4 Department of Pathology, Tata Memorial Center, Homi Bhabha National Institute, Mumbai, Maharashtra India; 5 Department of Pathology, University of Pittsburgh, Pittsburgh, Pennsylvania USA; 6 ACT Pathology, Canberra Health Services, Canberra, Australia; 7 School of Medicine and Psychology, ANU College of Science and Medicine, Canberra, Australia; 8 Department of Pathology, All India Institute of Medical Sciences, New Delhi, Delhi India; 9 Department of Pathology, Rigshospitalet, Copenhagen University Hospital, Copenhagen, Denmark; 10 The Fingerland Department of Pathology, University Hospital Hradec Kralove, Hradec Kralove, Czech Republic; 11 The Fingerland Department of Pathology, Charles University Faculty of Medicine in Hradec Kralove, Hradec Kralove, Czech Republic; 12 Department of Pathology, Rajiv Gandhi Cancer Institute and Research Centre, New Delhi, India; 13 Department of Pathology, NSW Health Pathology, Westmead Hospital, Sydney, NSW Australia; 14 Institute of Pathology, Faculty of Medicine, University of Ljubljana, Ljubljana, Slovenia; 15 Department of Pathology, Faculty of Medicine, Dalhousie University, Halifax, Nova Scotia Canada; 16 Department of Pathology and Immunology, Washington University School of Medicine, Saint Louis, MO USA; 17 Department of Anatomic and Molecular Pathology, Massachusetts General Hospital, Harvard Medical School, Boston, MA USA; 18 Department of Radiation Oncology, Princess Margaret Cancer Centre / University of Toronto, Toronto, ON Canada; 19 Department of Mathematics and Statistics, University of Sydney, Sydney, Australia; 20 Department of Pathology, Yonsei University College of Medicine, Severance Hospital, Seoul, Korea; 21 Tissue Pathology and Diagnostic Oncology, Royal Prince Alfred Hospital| NSW Health Pathology, Building 12, Missenden Road, Camperdown, NSW 2050 Australia; 22 University of Sydney, Central Clinical School, Royal Prince Alfred Hospital, Building 12, Missenden Road, Camperdown, NSW 2050 Australia

**Correction: Head and Neck Pathology (2025) 19:20** 10.1007/s12105-025-01753-7

The article “International Consensus Recommendations of Diagnostic Criteria and Terminologies for Extranodal Extension in Head and Neck Squamous Cell Carcinoma: An HN CLEAR Initiative (*Update* 1)”, written by Ruta Gupta, Timothy Fielder, Munita Bal, Simion I. Chiosea, Jane E. Dahlstrom, Aanchal Kakkar, Katalin Kiss, Jan Laco, Neha Mittal, Sunil Pasricha, Spinder Samra, Nina Zidar, Martin Bullock, Rebecca Chernock, William Faquin, Shao Hui Huang, Jean Yang and Sun Och Yoon, was originally published under exclusive license to Springer Science + Business Media, LLC, part of Springer Nature. As a result of the subsequent decision to publish the article under the open access model, the article’s copyright notice was changed on 28 February 2025 to © The Author(s) 2025 and the article is now distributed under a Creative Commons Attribution 4.0 International License, which permits use, sharing, adaptation, distribution and reproduction in any medium or format, as long as you give appropriate credit to the original author(s) and the source, provide a link to the Creative Commons licence, and indicate if changes were made. The images or other third party material in this article are included in the article’s Creative Commons licence, unless indicated otherwise in a credit line to the material. If material is not included in the article’s Creative Commons licence and your intended use is not permitted by statutory regulation or exceeds the permitted use, you will need to obtain permission directly from the copyright holder.

To view a copy of this licence, visit http://creativecommons.org/licenses/by/4.0.

